# Novel Bacterial Cellulose-Poly (Acrylic Acid) Hybrid Hydrogels with Controllable Antimicrobial Ability as Dressings for Chronic Wounds

**DOI:** 10.3390/polym10121323

**Published:** 2018-11-29

**Authors:** Clarence Chuah, Jing Wang, Javad Tavakoli, Youhong Tang

**Affiliations:** 1 Institute for Nanoscale Science and Technology, College of Science and Engineering, Flinders University, South Australia 5042, Australia; chua0131@flinders.edu.au (C.C.); jwang@flinders.edu.au (J.W.); javad.tavakoli@flinders.edu.au (J.T.); 2 Key Laboratory of Advanced Textile Composite Materials of Ministry of Education, Institute of Textile Composite, School of Textile, Tianjin Polytechnic University, Tianjin 300387, China; 3 Medical Device Research Institute, College of Science and Engineering, Flinders University, South Australia 5042, Australia

**Keywords:** poly (acrylic acid), bacterial cellulose, hydrogel, wound dressing, antimicrobial

## Abstract

This investigation examines the combination of poly (acrylic acid) (PAA) and bacterial cellulose (BC) nanofibers to synthesize hydrogel hybrid composites used for wound dressing application. Amoxicillin (AM) was also grafted onto the composites for drug release. Fourier transform infrared analysis and scanning electron microscopy conducted revealed the structure and porosity of the composite being developed, as well as the successful fabrication of BC-PAA composites. The results of mechanical testing and hygroscopicity revealed that the composite shows higher stability than hydrogels which are currently used worldwide, albeit with a slight reduction in swelling capabilities. However, the composite was revealed to be responsive to a rise in pH values with an increase in composite swelling and drug release. These results together with their morphological characteristics suggest that BC-PAA hydrogel hybrid composite is a promising candidate for wound dressing application.

## 1. Introduction

A wound that has failed to heal in an orderly and timely reparative process is the definition of a chronic wound, as they do not reach the anatomic and functional integrity required within three months [1,2]. Chronic wounds are a silent epidemic and they affect a significant portion of the global population, placing substantial cost and drain on healthcare systems and resources. Recent technological advancements have produced countless novel approaches to wound dressings, however chronic wounds remain an issue as their alkaline pH of 8 at the wound bed is conducive to bacterial bioburden and proteolytic activity [3].

Poly (acrylic acid) (PAA) is a common ionic polymer known for its pH-responsive behaviour as it is composed of polymeric backbones with ionic pendant groups [4]. In addition to that, hydrogels made from PAA are able to absorb and retain significant amounts of water within their structure without disintegrating. This pH-responsiveness on top of its water swelling characteristics allows hydrogel-based dressings to be used worldwide. However, their low mechanical strength makes the changing of hydrogel-based dressings difficult to handle [5].

On the other hand, bacterial cellulose (BC) fabricated by *Acetobacter xylinum* has remarkable mechanical properties as well as high crystallinity and water holding capacity [6]. This high tensile strength is due to its ultrafine nanofibrous network structure, making BC an attractive material for wound dressing application. Its biocompatible scaffold has also been used for other biomedical applications, such as blood vessels and scaffolds for tissue engineering amongst other applications [7,8].

Unfortunately, studies into the combination of these two materials are few, limited to ion-exchange membranes and drug-delivery systems [9,10]. Only one investigation has been conducted for burn wound healing in an animal model using the acrylic acid (AA) monomer [11]. However, infection needs to be prevented in chronic wounds, and BC has no antimicrobial activity to rely on. Hence, amoxicillin (AM) will be grafted onto the BC-PAA hydrogel hybrid composite to prevent wound infection. AM was used as it has a broad antimicrobial activity, bactericidal effect, and high therapeutic index, making it widely considered to be one of the most important antibiotics of the penicillin family [12].

In this study, a pH dual responsive biomaterial suitable for wound dressing application has been developed. The biomaterial being synthesized is that of a BC-PAA hydrogel hybrid composite, with AM being grafted onto the composite for drug release. It shows potential to be used as a dressing for chronic wounds.

## 2. Experimental Section

### 2.1. Materials

Acrylic acid (AA), amoxicillin (potency: ≥900 μg per mg) (AM), ammonium persulfate (APS), *N*-(3-Dimethylaminopropyl)-*N*′-ethyl carbodiimide hydrochloride (crystalline) (EDC), *N*,*N*′-Methylenebis (acrylamide) (MBA), *N*-hydroxysuccinimide (98%) (NHS) and phosphate buffered saline tablets (PBS) were purchased from Sigma-Aldrich, Sydney, Australia. Bacterial cellulose was purchased from Yide Foods Co. Ltd., Hainan, China. The potency for amoxicillin measures the drug activity by the amount required to obtain an effect of a given intensity, and Milli-Q water was used to prepare all aqueous solutions (18.2 MΩ cm at 25 °C).

### 2.2. Synthesis of AM-Grafted BC-PAA Hydrogel Hybrid Composite

BC with the size of 10 mm × 10 mm was cut and immersed into a previously prepared solution of 30 wt % AA (12 g AA dispersed in 28 cm^3^ Milli-Q water at 25 °C). 0.08 g MBA and 0.256 g APS powder were then added into the mixture before being ultrasonicated (Elmasonic S, Sydney, Australia) with the power of 37 kHz at 30 °C for 20 mins. The pieces were then placed onto a Petri dish and placed in an oven for 30 mins with 60 °C as its pre-set, increasing the temperature at a rate of 1 °C min^−1^ to 80 °C. Subsequently, 0.012 g NHS and 0.02 g EDC were added into 15 mL of PBS solution with 1 mg/mL AM concentration and stirred for 2 h. The BC-PAA specimens were then added and stirred gently at 20 rpm for 12 h at 25 °C before being freeze-dried last for 24 h at −40 °C.

The solution was to graft AM, an active molecule, onto the BC-PAA hydrogel hybrid composite. This synthesis method of grafting AM onto BC was conducted [13] in three steps comprising of: (i) grafting aminoalkylsilane groups onto BC through Si–O–C bonds utilising the chemical condensation method [14], (ii) –COOH of AM being treated with EDC/NHS, leading to NHS-activated ester groups being formed [15], and (iii) these groups on the AM reacting with the NH_2_ terminal group of BC, covalently linking AM onto the surface of BC through the amidation reaction [16].

### 2.3. Morphological Characterisation

The surface morphologies of BC-PAA hydrogel hybrid composite coated by a thin layer of platinum before being mounted on an aluminium stub was observed and evaluated by a scanning electron microscope (SEM, Inspect F50, FEI, Tokyo, Japan). The chemical bonds of BC, PAA, and AM grafted BC-PAA composites were investigated by Fourier-transform infrared spectroscopy (FTIR, Perkin Elmer Spectrum400, PerkinElmer, Waltham, MA, USA).

### 2.4. Mechanical Properties Test

A universal testing machine (Instron, Norwood, MA, USA) was used to perform tensile test and stress-strain analyses were calculated for rectangular specimens (L × W; 50 mm × 10 mm) of PAA and BC-PAA hydrogel hybrid composite. The thickness of each specimen was recorded at three different points before testing using a digital calliper after being mounted between the lower and upper wedge grips, with the averaged value being used for stress-strain calculations. As typical for hydrogels and soft tissues, an elongation rate of 5 mm/min was used for the various specimens [17]. A Bluehill 3 software recorded the force-elongation data and stress-strain diagrams were produced. Tests were conducted at room temperature in triplicate and averaged (95% CI), with specimens used only once before being discarded.

### 2.5. Hygroscopicity Test

The swelling kinetics of BC, PAA, and BC-PAA hydrogel hybrid composite were characterised by fully immersing the specimens in Milli-Q water at room temperature for 24 h, and the composite’s response to pH change was measured in PBS buffer solution by dissolving a PBS tablet (Sigma, Sydney, Australia) in 200 mL of Milli-Q water. pH values of 6, 7 and 8 were adjusted by using hydrochloric acid and sodium hydroxide accordingly. The change in swelling ratio for all specimens and different swelling environments were measured at specific time points until equilibrium using the equation:

$$SR=\frac{W_{t}-W_{i}}{W_{i}}\times 100,$$

where *W*_t_ and *W*_i_ are specimen weights at a time point and initial respectively. The average value (95% CI) of three measurements with standard deviation (SD) for each specimen was reported.

### 2.6. Drug Release Test

AM drug release of the BC-PAA hydrogel hybrid composite was obtained by immersing the specimen into 200 mL of PBS buffered solution at pH 6, 7, and 8 for 24 h. The solution was set in a water bath at 37 °C with magnetic stirring to ensure homogeneity of the solution. The concentration of AM being released from the specimen was measured using a Fluorescence Spectrophotometer (Cary Eclipse, Agilent, Santa Clara, CA, USA), exiting at 354 nm with emission being released at 445 nm. The intensity was measured against a calibration curve with known concentration of AM. All drug releases were performed in triplicate and the average reported.

## 3. Results and Discussion

### 3.1. Morphological Characterization

**Figure 1:**
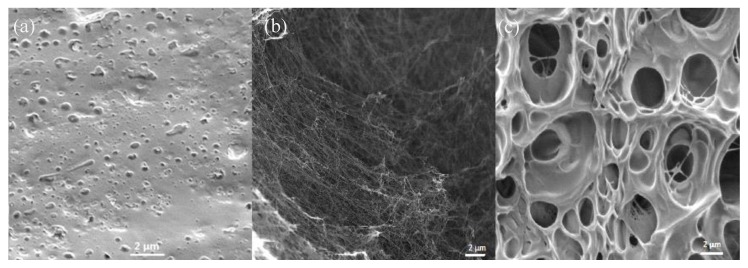
SEM images of (**a**) PAA, (**b**) BC, and (**c**) BC-PAA.

Figure 1 shows the SEM images of BC, PAA, and BC-PAA hydrogel hybrid composites. The morphology of PAA is shown in Figure 1a, exhibiting a sponge-like structure that seemingly facilitates water diffusion in all directions, making it suitable for would dressing applications. It is difficult to assign a single value to the size of the pore in Figure 1a as the porous structures are composed differently. Figure 1b shows the morphology of BC, observing crystalline nanofibers with 3D network structures, suggesting high over a large surface area. Figure 1c shows the BC-PAA hydrogel hybrid composite, observing a combination of the sponge-like structure of PAA and the crystalline nanofibers of BC. This suggests that PAA-BC composites have been successfully fabricated.

PAA-BC composites appear to have larger pores than PAA. This physical property is ideal and will result in a better wound dressing material than PAA as the larger pores allows for interconnectivity, enhancing in vitro cellular attachment and growth as well as promoting wound tissue infiltration and granulation tissue formation in vivo [18].

### 3.2. FTIR Analysis

**Figure 2:**
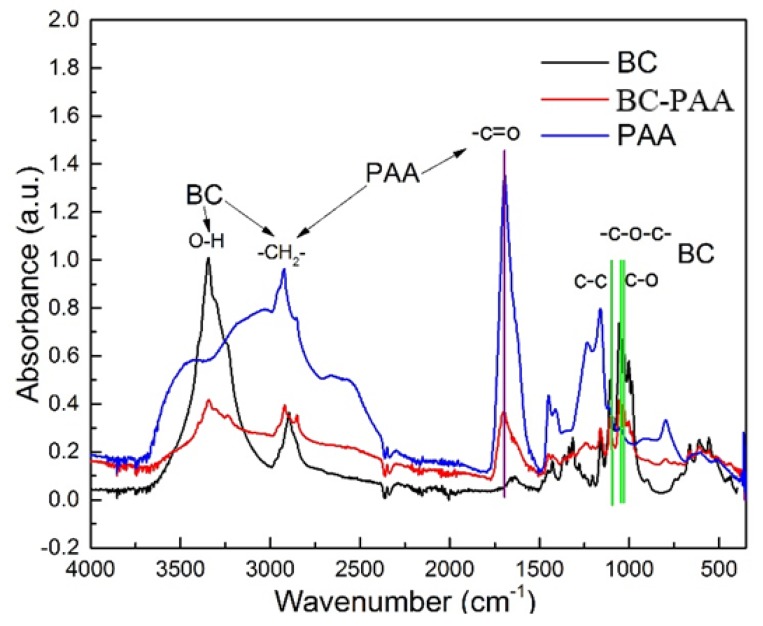
FTIR analysis of BC, PAA, and BC-PAA.

Figure 2 shows further evidence confirming the successful fabricating PAA-BC composite being revealed by FTIR analysis. For the IR spectra of the specimens, it can be seen that the characteristic peaks of BC and PAA all appear on the BC-PAA specimen. Between 3500 and 3250 cm^−1^, a broad band can be seen due to the intramolecular hydrogen bond and the hydroxyl group [19]. C–H stretch can be spotted peaking just before 3000 cm^−1^ [12]. The other main characteristic peaks observed on the FTIR spectra are at 1750 cm^−1^ (C=O stretching), 1163 cm^−1^ (C–O–C stretch of pyranose ring skeletal vibrations), and 1040 cm^−1^ (C–O asymmetric bridge stretching vibrations) [20].

### 3.3. Mechanical Properties Test

**Figure 3:**
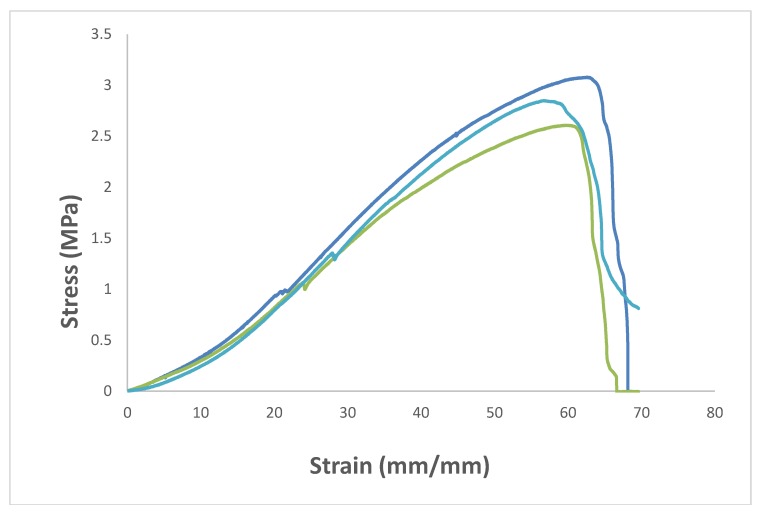
Mechanical properties of various BC-PAA composites.

The mechanical properties of BC-PAA hydrogel hybrid composite was compared against PAA hydrogel currently being used for wound dressing applications. Figure 3 displays the tensile test of the BC-PAA composite. As can be seen in Table 1, the tensile strength at break and the elongation at break of the pristine PAA specimen were 0.0619 ± 0.0079 MPa and 79.67 ± 7.13% respectively. For BC-PAA composite, the tensile strength at break increased to 2.85 ± 0.23 MPa, whilst the elongation at break was reduced to 57 ± 3.11%. This could be attributed towards the high mechanical properties of the BC nanofibers. Unfortunately, the drawbacks of BC having high mechanical properties would also mean that the nanofibers are more brittle in comparison to PAA hydrogel. However, the mechanical stability provided far outweighs the ductility of the BC-PAA composite as it allows for the hydrogel to be handled much more easily in comparison to just the PAA hydrogel alone.

### 3.4. Hygroscopicity Test

**Figure 4:**
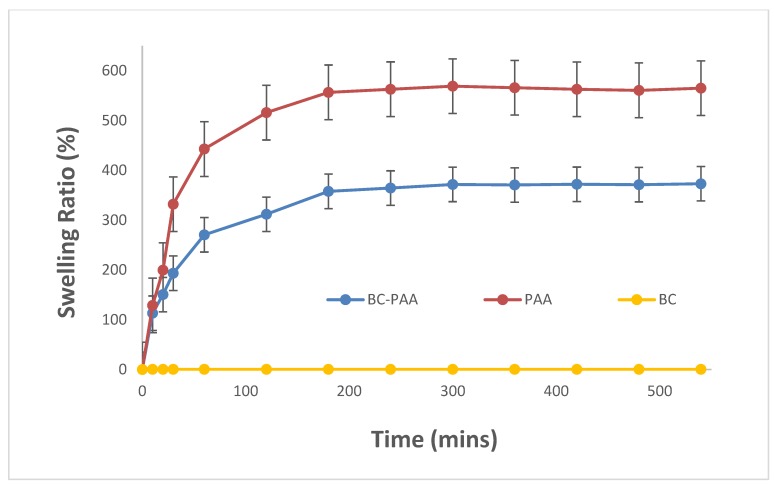
Swelling ratio of BC, PAA, and BC-PAA.

A critical and effective parameter for wound infection control is the wound dressing’s capability to swell as this relates the physico-chemical properties of the wound dressing to the biological properties of the wound [21]. The BC-PAA hydrogel hybrid composite significantly increased the swelling potential compared to BC alone, as shown in Figure 4. In contrast to PAA however, the BC-PAA hydrogel hybrid composite had a lower water swelling curve. This is because the addition of BC nanofibers limited the swelling ratio whilst providing the added mechanical stability. The plots of swelling ratio against time demonstrate that the absorption of water for PAA and BC-PAA specimens increased immensely at the beginning (0–120 min), before reaching equilibrium within 180 min. After that, the minute variation of PAA and BC-PAA specimens indicate excellent water retention properties suitable to maintaining the moist condition and humidity required for wounds to heal effectively.

**Figure 5:**
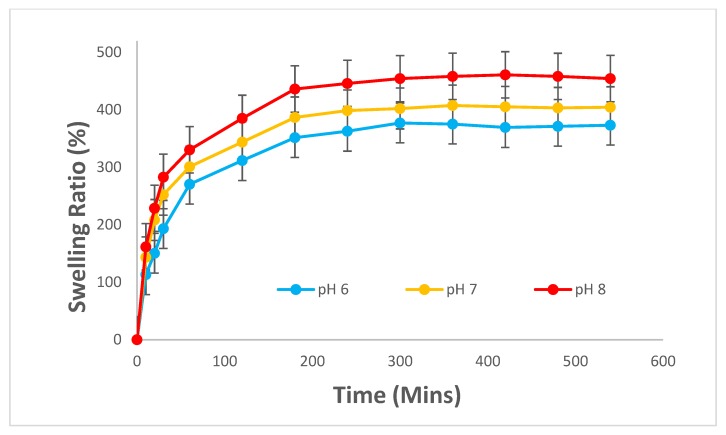
Swelling behaviour of BC-PAA at different pH values.

In addition, the BC-PAA hydrogel hybrid composite has shown to exhibit a response to pH change. According to Koetting et al, the kinetics of hydrogel swelling is largely determined by mass transfer limitation and that ionic gel swelling kinetics also rely on ion exchange, ion interactions, and Donnan equilibrium considerations [22]. As shown in Figure 5, the composite’s swelling ratio was low in an acidic medium (pH = 6), and increased as the pH increased to 8, an alkaline medium. The composite’s response to changes in pH could be due to the hydrogel’s structure, more specifically the carboxylic group’s hydrophilicity properties. The electrostatic repulsion causes the swelling of the hydrogel as AA’s carboxylic group gets deprotonated to a negatively charged carboxylate ion when the pH of the medium is increased [23,24]. This commends the suitability of the composite to be used as a wound dressing, as it demonstrates the capacity to absorb more water and potentially excess exudates at the higher pH end of chronic wound beds.

Despite this reduction in swelling property and elongation at breakpoints, PAA-BC is still a useful wound dressing material as its properties are comparable if not better than that of previous studies being conducted on wound dressing materials. These include other studies utilising bacterial cellulose [25,26], PVA hydrogel [27,28], chitin and chitosan biomaterials [29,30].

### 3.5. Drug Release Test

**Figure 6:**
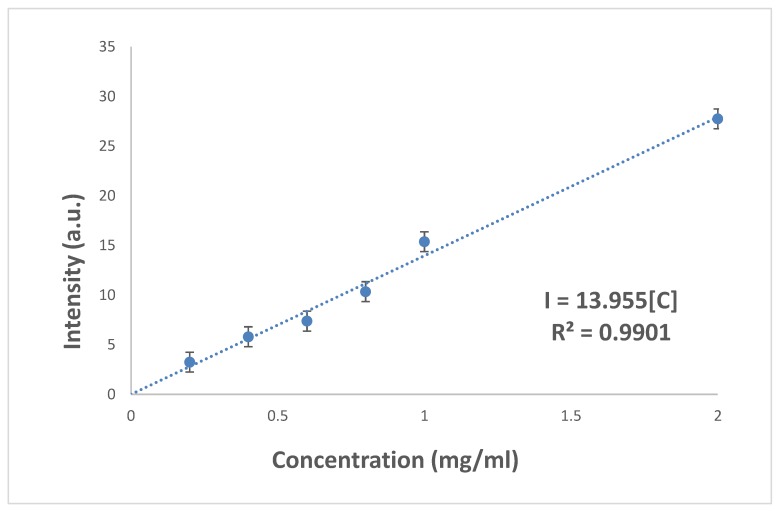
Calibration curve for amoxicillin drug release in PBS.

A calibration curve for the drug release of AM was conducted with known concentration to determine the intensity, as shown in Figure 6. This aids in identifying the concentration of AM being released by the BC-PAA hydrogel hybrid composite by comparing the intensity values to determine the concentration of AM being released.

**Figure 7:**
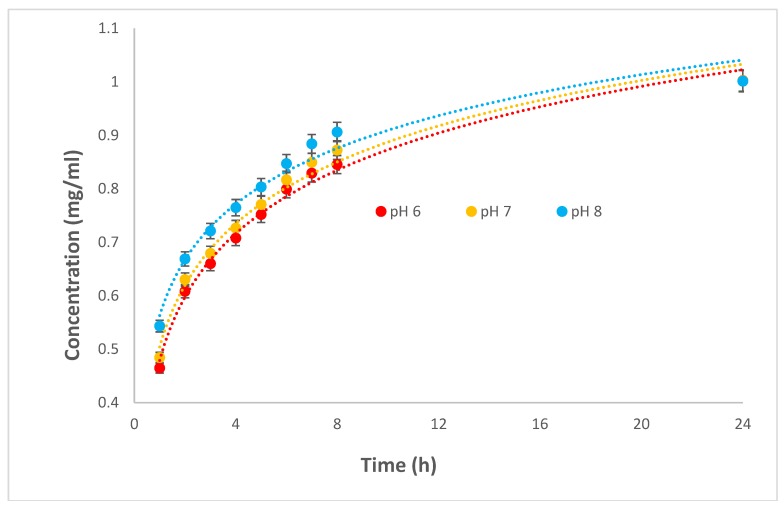
Amoxicillin drug release for BC-PAA composites in different pH values.

Figure 7 presents the AM drug release profiles of the BC-PAA hydrogel hybrid composite and its response in different mediums with pH values of 6, 7, and 8. The swelling medium penetrating the BC-PAA hydrogel hybrid composite seems to result in AM migrating from the network as the hydrogen bonding between AM and other components of the composite are weakened. It is known that the interaction of the drug with the polymer, the solubility of the drug in the release media, as well as the swelling behaviour of the hydrogel determines the drug release from a hydrogel [31]. AM was found to be released at the fastest rate from the composite at a pH medium of 8. The release of AM from the composite was lower at a pH medium of 6 as the acidic environment limited the swelling of hydrogel as discussed previously in the hygroscopicity test. For all pH mediums however, the immersion of the composite after 1 h showed a burst of AM being released. This was likely due to AM being present on the surface of the composite. Since drug release is being driven by the concentration gradient, the relatively high concentration gradient between the surface of the composite and the medium at initial immersion was likely the lead for the release of AM [4,32]. It took 24 h for all composites in respective pH mediums to release the grafted AM.

Statistical significance test performed has suggested that BC-PAA effectively releases drug at each different pH values more significantly during the latter half from 4–8 h than the former half from 1–4 h. This would imply that the dressing does not have a burst of amoxicillin at the start and differs with the change in pH values, but instead releasing the drug more rapidly in an alkaline pH over the course of the dressing being applied, of which is observed on the bed of chronic wounds.

According to the mechanical properties, hygroscopicity and drug release tests conducted alongside their sensitivity to pH, the BC-PAA composites stability is likely reflecting the probable hydrogen bonds being formed as well as physical entanglement at the interface between BC nanofibrous and PAA hydrogel. This is because the acrylic acid monomers were polymerized after penetration into the BC nanofibrous, paving the way for a physical entanglement to occur between the two biomaterials. In addition to that, hydrogen bonds could also have formed due to COOH– and OH– groups existing at the interface, increasing stability in the process. It seems that the mechanical and biomedical properties of the composites were improved because of the formation of hydrogen bonds along with physical entanglement of BC and PAA chains.

These results suggest that the BC-PAA hydrogel hybrid composite exhibited the potential to be a wound dressing with excellent response to a change in pH, that is, an increase in swelling capability as well as a faster release of AM at an alkaline pH of 8. This is especially relevant as a wound dressing for chronic wounds, where the pH fluctuates within the alkaline region of 7–8, conducive to bacterial bioburden and being more prone to bacterial growth and biofilm formation. Due to the faster release of AM in an alkaline environment, this composite would be able to release the needed antimicrobial aid required, as well as absorbing excess exudates being released by the chronic wound.

## 4. Conclusions

This investigation for the first time explored the potential of a biomaterial composite being fabricated by PAA and BC for wound dressing application. The FTIR analysis displayed the successful development of BC-PAA composites. Morphological analysis presented that the composite had a highly microporous sponge-like structure with BC nanofibers being present throughout the composite. The mechanical testing also displayed the strength of the BC-PAA composite when compared to the PAA hydrogel currently widely used as wound dressing.

The results of the hygroscopicity test suggested that the BC-PAA hydrogel hybrid composite had a response to a change in pH, with the swelling of the composite at an acidic pH medium of 6 being the least and an alkaline pH medium of 8 having the highest swelling ratio. This suggests that the composite would absorb more excess exudates at a higher pH, of which is known to be observed on wound beds of chronic wounds. Drug release from the composite also displayed a faster release at an alkaline pH medium of 8 in comparison to an acidic pH medium of 6, suggesting that drug release is also a response to the change in pH. The results obtained from this investigation imply that the BC-PAA hydrogel hybrid composite could be used as a wound dressing, such as that for chronic wounds. As such, further investigations are warranted under simulated conditions to examine the biocompatibility and biodegradability of this hydrogel composite.

## Figures and Tables

**Table 1:** Mechanical property comparisons of PAA and BC-PAA.

Specimens	Tensile Strength at Break (MPa)	Elongation at Break (%)
**PAA**	0.06 ± 0.01	79.67 ± 7.13
**BC-PAA**	2.85 ± 0.23	57.00 ± 3.11
